# A novel method to identify pathways associated with renal cell carcinoma based on a gene co-expression network

**DOI:** 10.3892/or.2015.4038

**Published:** 2015-06-08

**Authors:** XIYUN RUAN, HONGYUN LI, BO LIU, JIE CHEN, SHIBAO ZHANG, ZEQIANG SUN, SHUANGQING LIU, FAHAI SUN, QINGYONG LIU

**Affiliations:** 1 Department of Neurology, Shandong Provincial Hospital Affiliated to Shandong University, Jinan, Shandong 250021, P.R. China; 2 Department of Urology, Shandong Provincial Qianfoshan Hospital Affiliated to Shandong University, Jinan, Shandong 250014, P.R. China; 3 ICU, Affiliated Hospital of Jining Medical University, Jining, Shandong 272129, P.R. China; 4 Department of Urology, Jinan Central Hospital Affiliated to Shandong University, Jinan, Shandong 250013, P.R. China

**Keywords:** renal cell carcinoma, pathway, co-expression network, merge, rank

## Abstract

The aim of the present study was to develop a novel method for identifying pathways associated with renal cell carcinoma (RCC) based on a gene co-expression network. A framework was established where a co-expression network was derived from the database as well as various co-expression approaches. First, the backbone of the network based on differentially expressed (DE) genes between RCC patients and normal controls was constructed by the Search Tool for the Retrieval of Interacting Genes/Proteins (STRING) database. The differentially co-expressed links were detected by Pearson’s correlation, the empirical Bayesian (EB) approach and Weighted Gene Co-expression Network Analysis (WGCNA). The co-expressed gene pairs were merged by a rank-based algorithm. We obtained 842; 371; 2,883 and 1,595 co-expressed gene pairs from the co-expression networks of the STRING database, Pearson’s correlation EB method and WGCNA, respectively. Two hundred and eighty-one differentially co-expressed (DC) gene pairs were obtained from the merged network using this novel method. Pathway enrichment analysis based on the Kyoto Encyclopedia of Genes and Genomes (KEGG) database and the network enrichment analysis (NEA) method were performed to verify feasibility of the merged method. Results of the KEGG and NEA pathway analyses showed that the network was associated with RCC. The suggested method was computationally efficient to identify pathways associated with RCC and has been identified as a useful complement to traditional co-expression analysis.

## Introduction

Renal cell carcinoma (RCC) is a malignancy thought to arise from epithelial cells of the renal tubules and accounts for ~85% kidney cancers (1). The incidence of RCC has steadily shown a worldwide increase of 2–4% annually (2). Clinical manifestations of RCC are diverse and may lead to a range of non-specific and often misattributed symptoms (3). The classic triad of hematuria, flank pain and a flank mass has been suggested in only 10% of patients; however, >60% of the RCC are detected incidentally in patients not suspected of harboring a genitourinary malignancy (4). RCC has the highest mortality rate of the genitourinary cancers, as more than a third of patients with RCC are expected to succumb to the disease (5). Thus, identification of effective therapies and etiologic explanations of RCC is crucial.

The development of large scale of gene expression analysis has led to therapies at the gene level becoming more powerful and informative for the study of disease mechanism (6). For RCC, much has been accomplished since the identification of the Von Hippel-Lindau (*VHL*) in 1993 (7). *p53*, a tumor suppressor gene, when mutated inactivates the normal function of DNA damage surveillance (8). Additionally, some genes associated with RCC are typically detected through the analysis of many differentially expressed (DE) genes. The importance of these genes is evident in individual marker gene detection.

Despite the rich transcriptome data, identifying the disease mechanism involved remains a major challenge. Inconsistent results have been presented due to multiple issues of concern, including small sample size, measurement error and different statistical methods. The overlap is very low for the most significantly dysregulated genes across multiple studies (9). Based on the deficiency, a more effective means has been adopted by combining gene expression measurements over groups of genes that can be classified within common pathways. It identifies cancer markers by scoring known pathways by evaluating the coherency of genes expression changes (10). However, a large number of human genes have not yet been assigned to a definitive pathway based on pathway analysis. Network-based approaches particularly co-expression network offer an effective means to at least partially solve this challenge by providing potential malignancy diagnostic molecular and connecting them together. However, the results of the co-expression network are different when applying various constructed approaches, and there is a lack of methods to assess any reliable and comprehensive experimental data available.

In the present study, we created a novel method to integrate the gene-gene interaction correlations identified by a multiple co-expression network strategy, following a network-based pathway enrichment analysis. To achieve this, we first identified DE genes between RCC patients and normal controls using a linear Models for Microarray Data package, since we only focused on the shifted genes. The backbone of the co-expression networks was constructed using the Search Tool for the Retrieval of Interacting Genes/Proteins (STRING) database. Differentially co-expressed links were obtained based on the Pearson’s correlation score, empirical Bayesian (EB) approach and Weighted Gene Co-expression Network Analysis (WGCNA) based on DE genes of RCC. We ranked the gene pairs by the strength of their correlation for each method, and merged gene pairs by a rank-based algorithm. Furthermore, the pathway enrichment analysis based on the Kyoto Encyclopedia of Genes and Genomes (KEGG) database and the network enrichment analysis (NEA) method were performed to show the feasibility of the novel method.

## Materials and methods

### Identification of gene expression datasets and dataset preprocessing

Microarray expression profiles of RCC from Array Express with access no. E-GEOD-26574 (11), E-GEOD-36895 (12), E-GEOD-46699 (13) and E-GEOD-53757 (14), were selected to identify DE genes between RCC patients and normal controls. The four datasets were obtained from the Affymetrix GeneChip Human Genome U133 Plus 2.0 Array platform.

For each dataset, we applied standard methods to control the quality of gene microarray probe-level data (15). Briefly, to eliminate the effect of non-specific hybridization, background correction and quantile normalization were applied by the RMA method (16) and quantile-based algorithm (17). The quantile normalization method was a specific case of the transformation *x*′*_i_*=F^−1^(G (*x_i_*)), where *G* was estimated by the empirical distribution of each array and *F* using the empirical distribution of the averaged sample quantiles. The perfect match (PM) and mismatch (MM) values were revised using MAS algorithm (15), where the ideal MM would always be less than the corresponding PM and thus could be safely subtracted without risk of obtaining negative values. The summarization method was median polish (16). A multichip linear model was fit to the data from each probe set. In particular for the probe set *k* with *i*=1, …, *I_k_* probes and data from *j*=1,…, *J* arrays were fitted according to the model:

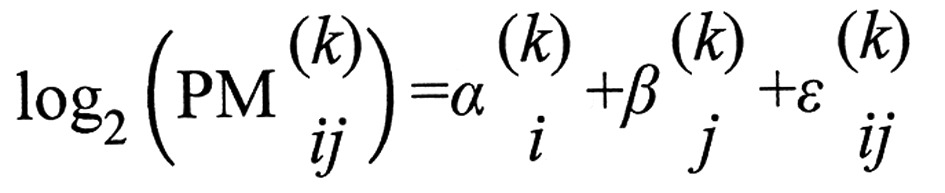
where *α_i_* was a probe effect and *β_j_* was the log_2_ expression value.

The data were subsequently screened by the feature filter method of the gene filter package, and the amount of genes with multiple probes was 20,109. The gene expression value for each gene was obtained, including 20,109 genes from 417 samples (179 normal controls and 238 RCC patients).

### Merging the multiple datasets

To calculate the co-expression value, it was necessary to merge all the independent data into a single dataset. Thus, the GenNorm method was applied to remove the unwanted batch effects in the gene expression values resulting from the use of different experimentation plans and methodologies in order to actually merge different datasets, as introduced by Taminau *et al* (18). The GenNorm method in an intuitive manner, which made datasets more comparable at z-score normalization and the expression values were calculated (19). The modified gene expression value $Y_{ij}^{k}$ was given by the expression:

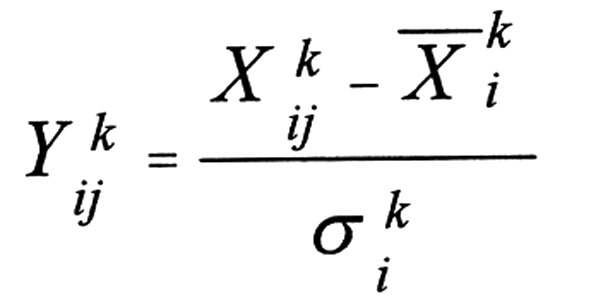
where *X_ij_* indicated each gene expression value in each study, ${\bar{X}}_{i}^{k}$ indicated mean gene expression value in the dataset, *K* indicated the number of studies and $\sigma_{i}^{k}$ indicated the standard deviation of the gene expression value.

The distribution of the combined data was inspected using a qualitative validation method to observe visually whether the samples from all the studies would cluster together or have a dataset-bias (20).

### Detection of DE genes

The linear Models for Microarray Data method was used to detect DE genes between RCC patients and normal controls based on 20,109 filtered genes. The P-values for all the genes were converted into the form of -log10 after being manipulated with t- and F-tests. Linear fit, empirical Bayes statistics and false discovery rate (fDR) correction were performed to the data by using Fit function (21). De genes were identified for further research with the threshold of P<0.05 and |log_2_FC| >2.

### Identification of gene-gene interaction correlations by multiple methods

Co-expression networks are instrumental for describing the pairwise relationships among the gene transcripts. Specifically, functionally related genes are frequently co-expressed across the samples. The co-expression network derived from the database and multiple co-expression approaches were considered a framework. The backbone of the network based on DE genes was constructed using the STRING database. Differentially co-expressed links were then detected by Pearson’s correlation, the EB approach and WGCNA.

### Construction of the backbone of the co-expression network using the STRING database

In this section, we investigated possible functional associations of DE gene pairs using the STRING database which provided a comprehensive, albeit quality-controlled collection of gene/protein associations for a large number of organisms with a global perspective (22). It is a carefully curated database that combines several different types of data. It comprises i) gene neighborhood and fusion, and phylogenetic profiles of the genomic context; ii) the co-occurrence and the co-expression of genes (i.e., variation of the transcript levels under the same conditions) by means of literature curation; iii) experimental evidence extracted from experimentally derived protein-protein interactions; iv) manually curated pathway databases; and v) text mining and homology in order to identify the co-mentioned genes (22). STRING assessed and integrated these data to obtain a confidence score for all protein/gene interactions. A sub-network was created using De genes, which was identified as mentioned in ‘Detection of DE genes’.

After assignment of the association scores, a final ‘combined score’ was computed between any pair of proteins. The combined scores were defined as the strength of the correlation and computed under the assumption of independence for the various sources, in a naive Bayesian manner. It was thus a simple expression of the individual scores (23):

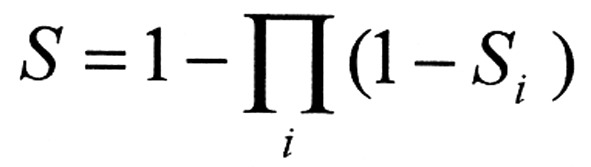


Evidence of the co-expression (individual scores) was chosen to describe the gene pairs with co-expression and the scores attributed by the STRING database between 0 and 1 to indicate the strength of the prediction were given.

### Construction of teh co-expression network using Pearson’s correlation test

In this study, we identified differentially co-expressed (DC) genes based on DE genes between RCC patients and normal controls using Pearson’s correlation test (24). PPIs weighted by the absolute average of Pearson’s correlation coefficients (PCCs) of the interacting gene pairs in the compared samples $\left(\left|{\bar{r}}_{E_{ij}}\right|\right),\left|{\bar{r}}_{E_{ij}}\right|$ and $\left|\Delta r_{E_{ij}}\right|$ were calculated:

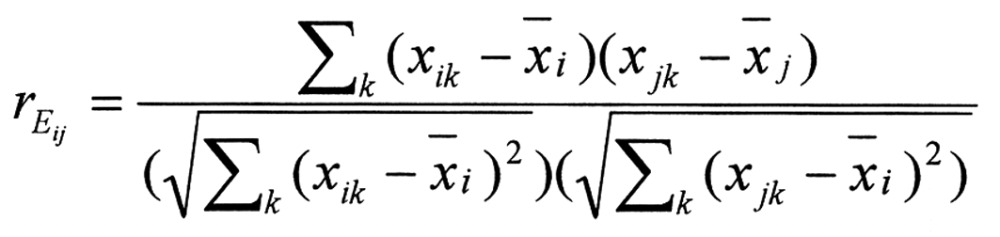
where *E_ij_* was the interactions between gene pairs *V_i_* and *V_j_*, *k* was the *kth* sample, *V_i_* and *V_j_* were ranked by their expression in the samples, respectively, and *X_jk_* was the rank of *V_i_* of the *kth* sample, *X_ik_* was the rank of *V_j_* of the *kth* sample, ${\bar{x}}_{i}$ and ${\bar{x}}_{j}$ were the average ranks of *V_i_* and *V_j_* in the samples, respectively.

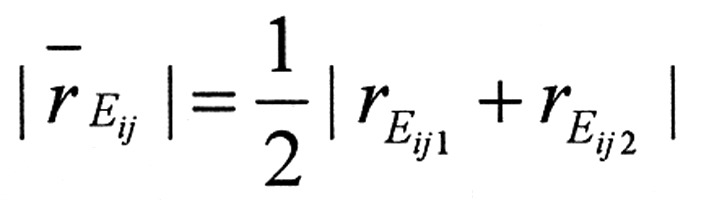


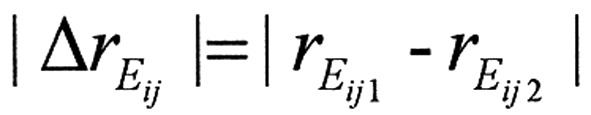
where *r_Eij_*_1_ and *r_Eij_*_2_ were the PCCs of *E_ij_* in the compared samples, respectively. Non-informative correlation pairs were filtered out with the half-thresholding strategy and a pair was kept in case the two PCCs had a q-value, where the q-value was an FDR estimated from the P<0.05 of PCC (25). We then defined $\left|\Delta r_{E_{ij}}\right|$ as the strength of the correlation in this method.

### Construction of co-expression network by the EB approach

A number of methods have been developed for co-expression analysis to identify DC gene pairs. However, these methods yield false findings under the conditions of large cardinality of the space to be interrogated (26). In this study, an effective approach of EB framework was conducted that provided an fDR controlled list of interesting pairs along with pair-specific posterior probabilities (27). The identification of DC gene pairs was processed at the following steps: three inputs of matrix X, the conditions array and the pattern object required. The expression values in an *m*-by-*n* matrix of X (where m indicated the number of genes/probes under consideration and n the total number of microarrays over all conditions) were normalized with background normalization and median correction and were generally represented on the log_2_ scale. The members of the conditions array with length *n* took values in 1,……, *K* (*K* indicated the total number of conditions). It was used to define the EC/DC classes with an ‘ebarraysPatterns’ object based on the unique values in the conditions array. Intra-group correlations for all p=m*(*m*-1)/2 gene pairs from X and the conditions array were calculated using bi-weight mid-correlation through the function makeMyD. The *p*-by-*K* of D matrix with correlations was obtained. The Mclust algorithm (28) was used to initialize the hyper parameters through the initializeHP function to detect the component in the normal mixture model that best fit the empirical distribution of correlations. The values of the component in the normal mixture model with component means, standard deviations and weights was used to initialize the expectation maximization (eM) algorithm (29). The three functions of the ‘full’, the ‘one-step’ and the ‘zero-step’ versions were different factors of the modified eM approach. In this step, the initial estimates of the hyper parameters rather than the ‘zero-step’ version were used to generate posterior probabilities of DC. After the eM computations were finished with the selected function, the prior diagnostic function for the prior predictive distribution was used to determine how well the model identified by the eM fit the data. The crit.fun function was used to provide a soft threshold by controlling the posterior probabilities of DC in order to identify particular types of DC gene pairs. The DC genes were distinguished from gene pairs having an invariant expression by controlling the posterior expected FDR at 0.05 and the co-expression network was constructed to represent the correlation between each pair of genes. In addition, we defined the DC as the strength of the correlation in this method.

### Construction of the co-expression network by WGCNA

WGCNA was frequently used to describe correlation patterns among gene expression profiles (30). For this method, the first step was to define a measure of similarity between the gene expression profiles. The *n*x*n* similarity matrix *S* = [*s_ij_*] was transformed into an *n*x*n* adjacency matrix A = [*a_ij_*] which encoded the connection strength between pairs of nodes. For each pair of genes *x_i_* and *x_j_* indicated similarity measured by *S_ij_*. we defined the absolute value of the Pearson’s correlations *S_ij_* = |*cor*(*x_i_*, *x_j_*)| of an unsigned network by employing a value between 0 and 1. However, a signed co-expression measure between *x_i_* and *x_j_* was applied to preserve the sign of the correlation which was defined with a simple transformation of the correlation:

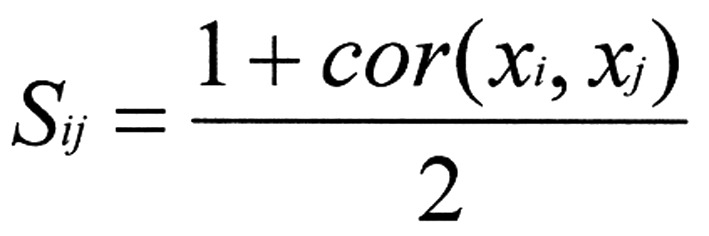


The adjacency function was used to determine the adjacency matrix A = [*a_ij_*]. The most widely used adjacency function was the signum function that implements a ‘hard’ threshold involving the threshold parameter τ:

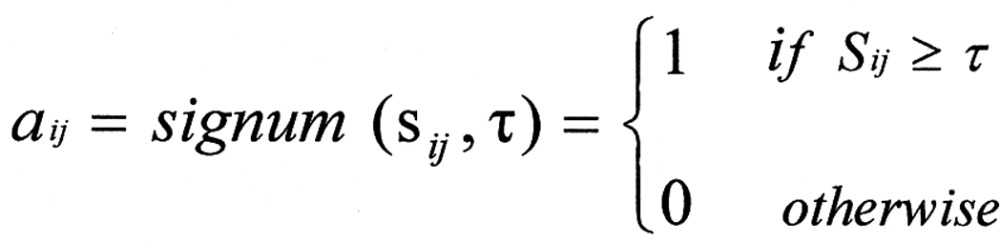


The hard threshold may lead to a loss of information; therefore a ‘soft’ adjacency function was needed. The power adjacency function was calculated as: *a_ij_* = |*s_ij_*|^β^ with the single parameter *β*.

As for the overlap of two nodes, which reflects their relative interconnectedness, the topological overlap matrix (ToM) Ω = [*ω_ij_*] provided a similarity measure. To turn it into a dissimilarity measure, it was subtracted from one, i.e, the topological overlap based dissimilarity measure was defined by $d_{ij}^{\omega }=1-\omega_{ij}.$ In addition, we defined the weight value as the strength of the correlation in this method.

### Merging of co-expressed gene pairs

Determination of the significance of the changes occurring and the number of selected gene pairs likely to be truly differentially co-expressed is important. After gene co-expression was analyzed using the above four methods, the score of each co-expressed gene pair was obtained. Considering the results were different due to utilizing various approaches, all the score values were assessed further to ensure their uniformity and converted in the form of rank/(total number of gene pairs) based on the Rank Products (RP) algorithm (31).

The RP-values were calculated over all the possible pairwise comparisons. The algorithm *i* (*i* = STRING database, Pearson’s correlation, EB approach and WGCNA), each examining *n* gene pairs were considered, whereby the RP for a certain gene pair *g* would be:

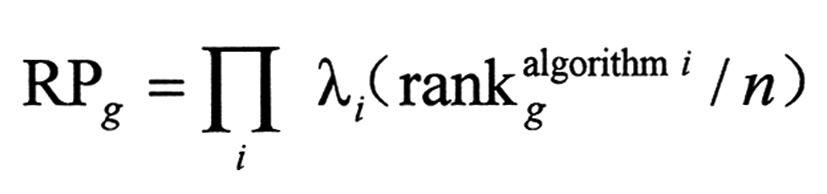


This was potentially interpreted as a P-value (=RP-value), as it described the probability of observing gene pair *g* at a certain rank (rank^algorithm^ *^i^*), with *λ_i_* being the weight coefficient of each algorithm. If the gene pairs were not differentially co-expressed in any method, the weight value of pairwise in this method would equal to 1.

Subsequently, for each gene pair *g*, a conservative estimate of the percentage of false-positives (PFP) was calculated when this gene pair (and all the gene pairs with RP-values smaller than this cut-off value) was considered as significantly differentially co-expressed: *q_g_* = *E*(RP*_g_*)/rank(*g*). In the present study, rank(*g*) denoted the position of gene pair *g* in a list of all the gene pairs sorted by the increasing RP-value, i.e., it was the number of gene pairs accepted as significantly regulated. This estimated the FDR and extended the list of accepted gene pairs up to the gene pair with a *q_g_*-value of <0.1. The DC gene pairs were therefore obtained for subsequent study.

### Pathway enrichment analysis

To verify the feasibility of the merged method, the pathway enrichment analysis of DC gene pairs based on the KEGG database and NEA method were performed in this study.

### KEGG database

To investigate the biological functions of the DE genes, KEGG pathway enrichment analysis was performed by Database for Annotation, Visualization and Discovery (DAVID) (32). KEGG pathways with P<0.01 were chosen based on the Expression Analysis Systematic Explorer (EASE) test applied in DAVID. EASE analysis of the regulated genes indicated molecular functions and biological processes unique to each category (33). The EASE score was used to detect the significant categories. The threshold of eASe score <0.01 and the minimum number of genes for the corresponding term >2 were considered significant for a category.

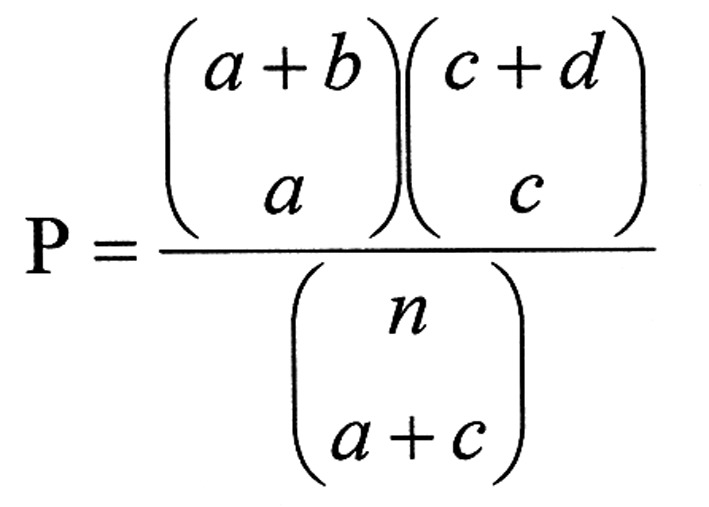
where *n* (*n* = *a*′*+b+c+d*) was the number of background genes, *a*′ was the gene number of one gene set in the gene lists, *a*′ + *b* was the number of genes in the gene list including at least one gene set, *a*′ + *c* was the gene number of one gene list in the background genes and *a’* was replaced with *a* = *a*′-1.

### Network enrichment analysis method

A NEA method, which systematically implemented the network approach to describe novel gene sets with biologically meaningful functional categories, was selected to analyze DC gene pairs of RCC (34). A fast network randomization algorithm was used in the method to obtain the distribution of any network statistics under the null hypothesis of no association between an altered gene sets (AGS) and functional gene sets (FGS) (35).

To investigate the functional heterogeneity of individual RCC, we ranked the differences between individuals. The differential expression of gene *g* in patient *i* compared to the group of patients was calculated as: Difference values = log(*T/N*) *ig* − ave(log(*T/N*)).*_g_*, where log(T/N) was the log intensity-ratio of RCC vs. normal expression. Let *A*(*k*) was an AGS of size *k*, and define *n_AF_*(*k*), a measure of network connectivity between *A*(*k*) and a known FGS (*F*), as the number of links between members of *A*(*k*) and *F*. Since the connectivity was dependent on the constituent genes, we corrected *n_AF_*(*k*) by its expected value: *d_AF_*(*k*) = *n_AF_*(*k*)−*μ_AF_*(*k*), where *μ_AF_*(*k*) was the expected number of links between A(*k*) and *F*.

## Results

### Identification of the DE genes

In total, 20,109 genes in E-GEOD-26574, E-GEOD-36895, E-GEOD-46699 and e-GeoD-53757 were identified by reading the gene expression profiles using an Affy package. After preprocessing of the expression profile dataset, we obtained 753 De genes between RCC patients and normal controls with the thresholds of P<0.05 and |log_2_FC| >2.

### Analysis of co-expression networks

In the present study, the co-expression networks of 753 DE genes were constructed by four methods (STRING database, Pearson’s correlation tests, EB approach and WGCNA). We achieved a co-expressed relationship between gene and gene or co-expressed gene pairs and scores of gene pairs.

**Figure 1:**
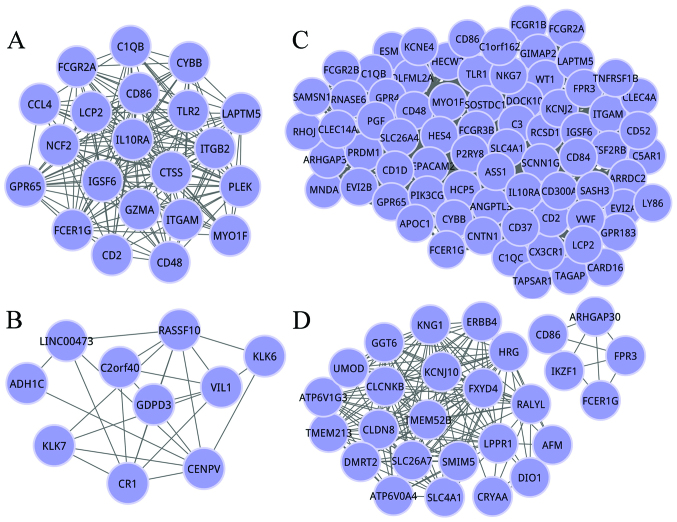
Co-expression networks based on hub genes of RCC from co-expression networks constructed by the (A) STRING database, (B) Pearson’s correlation tests, (C) eB method and (D) wGCNA. Genes (nodes) are connected by edges if their vectors are sufficiently similar. Black edge is associated with a pair of genes under thresholds. RCC, renal cell carcinoma; STRING, Search Tool for the Retrieval of Interacting Genes/Proteins; EB, empirical Bayesian; WGCNA, Weighted Gene Co-expression Network Analysis.

We obtained 842 co-expressed gene pairs of RCC based on the STRING database. According to the Pearson’s correlation tests, 371 co-expressed gene pairs were obtained. We achieved 2,883 co-expressed gene pairs dependent on the EB method with the threshold fDR ≤0.05. when constructing the co-expression network of DE genes using the WGCNA method, 1,595 co-expressed gene pairs were obtained. The number of co-expressed gene pairs based on the EB approach was higher than that of the other three methods. The genes at the top of degree distribution (≥90% quantile) in the significantly perturbed co-expression networks were defined as hub genes. The co-expression networks of hub genes from the four methods are shown in Fig. 1.

### Merging of the co-expressed gene pairs

**Figure 2:**
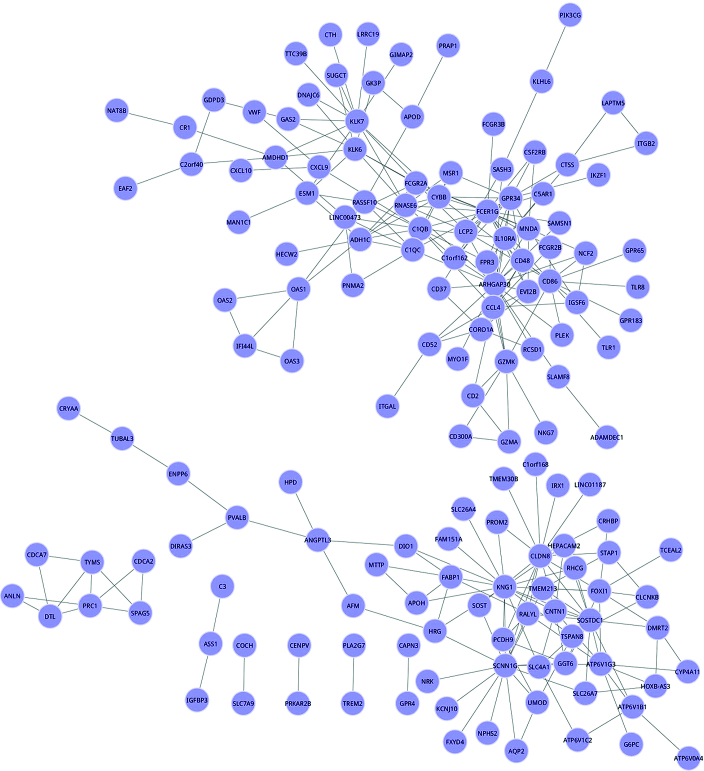
Co-expression network of 281 DC gene pairs of RCC from the merged matrix. Genes (nodes) are connected by edges if their vectors are sufficiently similar. Black edge is associated with a pair of genes with q-value correction (P<0.1). DC, differentially co-expressed; RCC, renal cell carcinoma.

We merged all the co-expressed gene pairs identified from the four methods utilizing RP algorithm, and 13,945 genes were assessed after merging. Two hundred and eighty one DC gene pairs were obtained after q-value correction (P<0.1) and their co-expression network is shown in Fig. 2. There were 154 nodes and 281 edges in the co-expression network.

### Pathway enrichment analysis

For the KEGG pathway enrichment analysis, our results showed that 753 DE genes were significantly enriched in 130 terms. Co-expressed gene pairs obtained from the four methods and 281 DC gene pairs were enriched in pathways, with the cytokine-cytokine receptor interaction and systemic lupus erythematosus being common pathways of the five types of co-expressed gene pairs. Thirteen pathways were obtained from 281 DC gene pairs (Table I). Counts of cytokine-cytokine receptor interaction, chemokine signaling pathway, cell adhesion molecules, toll-like receptor signaling pathway and the neuroactive ligand-receptor interaction were increased by 10.

The NEA method was performed on 281 DC genes using a neaGUI package in R (Table II). The metabolic pathways had the highest number links with 1,282, the following was phagosome, chemokine signaling pathway, cell adhesion molecules and natural killer cell-mediated cytotoxicity.

## Discussion

In the present study, co-expression networks were constructed using the STRING database, Pearson’s correlation tests, EB method and WGCNA method. We merged these co-expressed gene pairs together using RP algorithm and scored 281 DC gene pairs. The KEGG pathway enrichment analysis and NEA method were selected to verify the feasibility of this merged method. The results show that cytokine-cytokine receptor interaction and metabolic pathways were the most significant biological processes that were closely associated with RCC.

Diagnostic or prognostic markers were usually obtained by identification of the most significant DE genes in the high-throughput case-control studies of a disease. However, previous findings have shown that the most significant De genes obtained from different studies for a particular cancer are typically inconsistent (36). To overcome this problem, significant genes and biological processes were assessed for disease-association using a network strategy, particularly the co-expression network (37). When constructing a co-expression network, the STRING database is the most commonly used method. Of note is that a few other approaches have been developed for co-expression analysis, such as the Pearson’s correlation tests, EB approach and WGCNA.

There are some drawbacks to employing these methods (38). For the STRING database, the networks, which are supposed to be static, may not reflect the specific condition of the individuals or specific disease. For the Pearson’s correlation tests, all possible variations are measured although are the effects on gene expression not considered, thereby producing many false-positive results. The EB approach examines network variations and their effects on gene expression. Nevertheless a disease-associated gene may lead to the differential expression of its interacting genes even if there is no network rewiring in certain situations. WGCNA suggested a tight network that was closer to properties of small networks in a general framework as compared to the Pearson’s correlation tests.

Therefore, we developed a new method by merging co-expressed gene pairs together to overcome these problems based on an RP algorithm. In our merged method, weight value was utilized to reflect the differential importance of each method, and the weight of each dataset was set equally since we treated all the datasets equally. If certain reliable properties serve as the backbone, a decrease in the weight value is merely required. For instance, in the given database, if it occupied the dominant position, we would select 0.1 as its weight value and 1 for the remaining methods. The merged method provides a straightforward and statistically stringent means to determine the significance level for each gene pair, allowing for the flexible control of the false-detection rate and familywise error rate in the multiple testing (31).

Results of the merged method showed that the cytokine-cytokine receptor interaction and metabolic pathways were the most significant biological processes of RCC. Cytokines that were crucial intercellular regulators mobilized cells engaged in innate as well as adaptive inflammatory host defenses, cell growth and cell death (39). Cytokine receptors functioned to inhibit tumor development and progression in response to infection, inflammation and immunity. A more detailed understanding of cytokine-tumor-cell interactions provided new opportunities for improving cancer immunotherapy, such as RCC (40). It has been reported that the tumor response in treatment-naive and cytokine-pretreated patients is associated with advanced metastatic RCC (41). Therefore, the cytokine-cytokine receptor interaction pathway was closely associated with RCC, suggesting that the merged method was feasible.

Linehan *et al* found that mutations in each of kidney cancer genes resulted in dysregulation of metabolic pathways, suggesting that kidney cancer is a disease of cell metabolism (42). In addition, metabolic activities in proliferating cells are fundamentally different from those in non-proliferating cells, and are associated with signal transduction pathways and transcriptional networks of RCC (43). The essential hallmarks of cancer were intertwined with an altered cancer cell-intrinsic metabolism. Additionally, the constitutive activation of signaling cascades that stimulate cell growth has a profound impact on the anabolic metabolism (44). Thus cancers, for example RCC, were closely associated with cell metabolism.

In conclusion, we created a novel merged method to identify genes and pathways associated with RCC, and the KEGG and NEA pathway analyses have shown the correctness and feasibility of this method. The recommended method is computationally efficient to identify genes and pathways of RCC and has been proven to be a useful complement to traditional co-expression analysis.

## Figures and Tables

**Table I:** KEGG pathways based on 281 DC gene pairs.

Term	Counts
Cytokine-cytokine receptor interaction	24
Chemokine signaling pathway	16
Cell adhesion molecules (CAMs)	14
Toll-like receptor signaling pathway	13
Neuroactive ligand-receptor interaction	12
Systemic lupus erythematosus	10
Natural killer cell-mediated cytotoxicity	9
Oxidative phosphorylation	8
Aldosterone-regulated sodium reabsorption	8
*Vibrio cholerae* infection	7
Epithelial cell signaling in *Helicobacter pylori* infection	6
Lysosome	5
Fc ε RI signaling pathway	3

KEGG, Kyoto Encyclopedia of Genes and Genomes; DC, differentially co-expressed.

**Table II:** Results of NEA based on 281 DC gene pairs.

Path_name	No. of links
Metabolic pathways	1,282
Phagosome	848
Chemokine signaling pathway	800
Cell adhesion molecules (CAMs)	701
Natural killer cell-mediated cytotoxicity	653
Osteoclast differentiation	609
Cytokine-cytokine receptor interaction	603
Leishmaniasis	578
Toxoplasmosis	563
Regulation of actin cytoskeleton	554
Fc γ R-mediated phagocytosis	529
Leukocyte transendothelial migration	529
*Staphylococcus aureus* infection	502
Rheumatoid arthritis	501
Neuroactive ligand-receptor interaction	489
Pathways in cancer	480
Viral myocarditis	479
Antigen processing and presentation	438
T-cell receptor signaling pathway	438
Systemic lupus erythematosus	411
Hematopoietic cell lineage	406
Fc ε RI signaling pathway	388
B cell receptor signaling pathway	382
Endocytosis	373
Autoimmune thyroid disease	356
Graft-vs.-host disease	354
Type I diabetes mellitus	351
Allograft rejection	347
Intestinal immune network for IgA production	344
Focal adhesion	327
Jak-STAT signaling pathway	318
Toll-like receptor signaling pathway	311
Chagas disease (American trypanosomiasis)	289
Calcium signaling pathway	263
Asthma	254
Amoebiasis	253
Olfactory transduction	251
Neurotrophin signaling pathway	251
Lysosome	248
Cell cycle	247
Bacterial invasion of epithelial cells	237
VEGF signaling pathway	231
Purine metabolism	225
Primary immunodeficiency	222
Hepatitis C	221
RNA transport	219
Pathogenic *Escherichia coli* infection	216
Oocyte meiosis	204
Epithelial cell signaling in *Helicobacter pylori* infection	203
Drug metabolism-cytochrome P450	194
Shigellosis	190
Pyrimidine metabolism	188
Spliceosome	185
Protein processing in endoplasmic reticulum	184
Axon guidance	184
Pancreatic cancer	180
Metabolism of xenobiotics by cytochrome P450	177
Cytosolic DNA-sensing pathway	173
Adherens junction	168
Huntington’s disease	160
Retinol metabolism	158
Drug metabolism - other enzymes	157
Apoptosis	157
Complement and coagulation cascades	156
Pancreatic secretion	156
Colorectal cancer	155
Wnt signaling pathway	154
*Vibrio cholerae* infection	154
Arachidonic acid metabolism	147
Alzheimer’s disease	146
Malaria	146
Small cell lung cancer	144
Long-term depression	143
Oxidative phosphorylation	142
Phosphatidylinositol signaling system	141
NOD-like receptor signaling pathway	138
Acute myeloid leukemia	133
Non-small cell lung cancer	133
DNA replication	130
Salivary secretion	125
Steroid hormone biosynthesis	118
Starch and sucrose metabolism	118
Dilated cardiomyopathy	117
Ubiquitin-mediated proteolysis	114
Amyotrophic lateral sclerosis (ALS)	114
mRNA surveillance pathway	110
Melanogenesis	109
Hypertrophic cardiomyopathy (HCM)	106
Glycerophospholipid metabolism	105
Type II diabetes mellitus	104
Carbohydrate digestion and absorption	103
Porphyrin and chlorophyll metabolism	102
Glutathione metabolism	100
Linoleic acid metabolism	97
Ribosome biogenesis in eukaryotes	97
Other types of O-glycan biosynthesis	96
Adipocytokine signaling pathway	96
Aldosterone-regulated sodium reabsorption	95
Long-term potentiation	94
p53 signaling pathway	93
Pentose and glucuronate interconversions	92
RNA degradation	91
Inositol phosphate metabolism	88
Bile secretion	88
Ascorbate and aldarate metabolism	86
Prion diseases	86
eCM-receptor interaction	82
Collecting duct acid secretion	82
Arginine and proline metabolism	79
Proteasome	78
Parkinson’s disease	78
Nucleotide excision repair	75
TGF-β signaling pathway	74
Fat digestion and absorption	74
Ether lipid metabolism	70
Ribosome	69
Protein digestion and absorption	68
Mismatch repair	67
PPAR signaling pathway	66
African trypanosomiasis	65
Alanine, aspartate and glutamate metabolism	59
Base excision repair	58
Aminoacyl-tRNA biosynthesis	53
Citrate cycle (TCA cycle)	52
RNA polymerase	51
α-linolenic acid metabolism	49
Glycerolipid metabolism	47
Tryptophan metabolism	45
Glycine, serine and threonine metabolism	44
Proximal tubule bicarbonate reclamation	43
Tyrosine metabolism	41
Homologous recombination	40
Notch signaling pathway	40
N-Glycan biosynthesis	37
Protein export	37
Bladder cancer	36
Histidine metabolism	33
Phenylalanine metabolism	31
Cardiac muscle contraction	30
Hedgehog signaling pathway	30
Other glycan degradation	29
Pantothenate and CoA biosynthesis	27
Nitrogen metabolism	25
Thyroid cancer	24
β-alanine metabolism	22
Renin-angiotensin system	22
Vitamin digestion and absorption	19
Valine, leucine and isoleucine biosynthesis	17
Glyoxylate and dicarboxylate metabolism	17
Non-homologous end-joining	17
Phenylalanine, tyrosine and tryptophan biosynthesis	16
Taurine and hypotaurine metabolism	16
Circadian rhythm-mammal	16
Basal cell carcinoma	15
Butanoate metabolism	13
Folate biosynthesis	13
Caffeine metabolism	12
Terpenoid backbone biosynthesis	11
Biosynthesis of unsaturated fatty acids	9
Basal transcription factors	8
Riboflavin metabolism	7
Fatty acid elongation in mitochondria	2
Glycosylphosphatidylinositol (GPI)-anchor biosynthesis	2
Mucin type O-glycan biosynthesis	1

NEA, network enrichment analysis; DC, differentially co-expressed.
